# Regulation of Iron Homeostasis and Use in Chloroplasts

**DOI:** 10.3390/ijms21093395

**Published:** 2020-05-11

**Authors:** Gretchen E. Kroh, Marinus Pilon

**Affiliations:** Department of Biology, Colorado State University Department of Biology, Fort Collins, CO 80523, USA; gkroh@colostate.edu

**Keywords:** photosynthesis, iron homeostasis, green lineage, Fe–S, plants, Cyanobacteria, Chlamydomonas, chloroplast

## Abstract

Iron (Fe) is essential for life because of its role in protein cofactors. Photosynthesis, in particular photosynthetic electron transport, has a very high demand for Fe cofactors. Fe is commonly limiting in the environment, and therefore photosynthetic organisms must acclimate to Fe availability and avoid stress associated with Fe deficiency. In plants, adjustment of metabolism, of Fe utilization, and gene expression, is especially important in the chloroplasts during Fe limitation. In this review, we discuss Fe use, Fe transport, and mechanisms of acclimation to Fe limitation in photosynthetic lineages with a focus on the photosynthetic electron transport chain. We compare Fe homeostasis in Cyanobacteria, the evolutionary ancestors of chloroplasts, with Fe homeostasis in green algae and in land plants in order to provide a deeper understanding of how chloroplasts and photosynthesis may cope with Fe limitation.

## 1. Introduction

**Figure 1:**
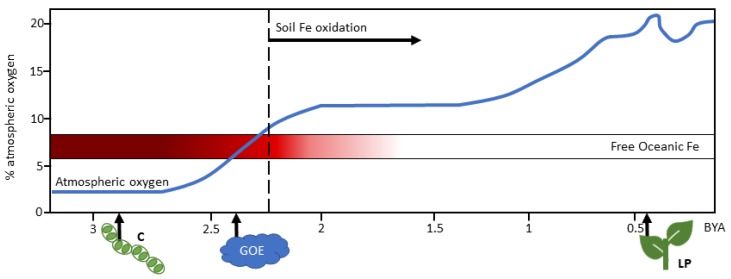
Conceptual model of relationship between atmospheric oxygen and Fe availability during Earth’s history. Percent atmospheric oxygen is presented on the y-axis with time on the x axis. The early earth’s atmosphere contained about 0.001% oxygen until oxygenic photosynthesis began in Cyanobacteria about 2.7 billion years ago (BYA). The increase in oxygen formation led to lowered availability of Fe. Banding iron formations in strata are thought to represent times of intermittent Fe oxide formation from free oceanic Fe as atmospheric oxygen began to increase. Banding iron formations are not found prior to 3 BYA, suggesting the low oxygen content allowed for free Fe. Banding iron formation frequency peaks around 2.5 BYA and these are again not found following 1.8 BYA suggesting that Fe oxidation became constant. The start of soil Fe oxidation is dated to around 2.3 BYA. Timeline depicts billions of years ago. Blue line represents the general trend of increasing oxygen to current levels. Red gradient represents estimated amount of free Fe^2+^ based on frequency of banding Fe patterns in strata with darker red representing more free Fe^2+^. Dotted line represents onset of soil Fe oxidation in the geological record. First occurrence of Cyanobacteria (C), the Great Oxygen Event (GOE) and land plants (LP) are noted along the x axis. Trends are based on data reviewed in [3,9].

In the early, anoxic, reducing environment of Earth, life was built on iron (Fe). In the low oxygen environment, the bioavailable form of Fe, Fe^2+^, was abundant and readily reacted with sulfur (S) to form pyrite (Fe_2_S). Early life began to incorporate pyrite into biochemistry for catalytic functions such as electron transport [1]. Fe–S clusters were especially important in the evolution of the oxygenic photosynthetic electron transport chain [2]. After oxygenic photosynthesis evolved, atmospheric oxygen levels began to increase from less than 0.001% to 21% [3]. The oldest fossils of Cyanobacteria—the earliest contributors to oxygenic photosynthesis—are dated to 2.7 billion years ago (BYA) [3]. The rise in oxygen in the Earth’s atmosphere is estimated to have begun about 2.4 BYA (Figure 1). However, modern oxygen levels are thought to be the result of land plant evolution 420–400 million years ago (Figure 1) [4]. The rise of oxygenic photosynthesis resulted in geological shifts where the poorly bioavailable, oxidized Fe^3+^ now accumulates [2]. 

While Cyanobacteria are considered to be the earliest oxygenic photosynthetic organisms, endosymbiosis of a Cyanobacterial cell allowed expansion of photosynthesis to a eukaryotic lineage, giving rise to Archaeplastida, which includes land plants [5,6]. The predominance of oxidized Fe presents a major problem for most organisms, but even more so for modern photosynthetic organisms, as the photosynthetic electron transport chain has an exceptionally high demand for Fe, requiring an estimated 28 Fe atoms per chain [7]. In general, the chloroplast is a strong sink for Fe and contains 60%–80% of total Fe in leaves [8]. 

Fe deficiency results in a lack of activity in Fe-requiring pathways, such as photosynthetic electron transport, cofactor assembly, and sulfur and nitrogen metabolism, which compromises an organism’s growth [10]. During Fe deficiency, expression of specific reactive oxygen species (ROS)-scavenging molecules is up- or down- regulated, suggesting a need to prevent potential ROS-induced damage [11]. The potential accumulation of ROS may arise from impairment of photosynthetic electron transport as a result of Fe deficiency [12,13]. In stress conditions when electron transport is impaired, the organism must adapt to scavenge harmful ROS that may result from excess excitation of photosystems [14]. During Fe deficiency, mechanisms to increase Fe uptake and economize Fe for most important cellular functions must be employed. Thus, Fe deficiency, while more common in nature than Fe excess, may also have a greater impact on an organisms’ health. 

The strong requirement for Fe in photosynthesis has led to the evolution of mechanisms in photosynthetic organisms to minimize stress from Fe deficiency. When Fe is limiting, photosynthetic organisms can respond by (1) increasing Fe uptake; (2) remodeling metabolism; (3) increasing efficiency of Fe utilization; (4) remobilization of stored Fe. For example, several Cyanobacteria and the unicellular eukaryotic algae, such as *Chlamydomonas reinhardtii*, can increase Fe uptake on the cell membrane [15,16,17]. Land plants must regulate increased Fe uptake at the root epidermis, and also mediate Fe transport into the xylem from the pericycle for Fe translocation to shoots [18,19,20,21]. 

It is essential that photosynthetic organisms respond to changes in Fe status to maintain photosynthesis and chloroplast integrity without aberrant production of ROS resulting from the presence of free Fe atoms and decreased photosynthetic performance. As Cyanobacteria are closely related to the earliest ancestors of chloroplasts, their responses to Fe deficiency may provide insight into chloroplast Fe homeostasis and regulation. Chlamydomonas and land plants share a common, chloroplast-containing ancestor. Therefore, comparative analyses between Chlamydomonas and land plants can uncover conserved, ancestral strategies for responding to Fe deficiency. Similarities in Fe deficiency responses across green lineages (Chlorophyte and Streptophyte lineages [22]) have given insight into the evolution of the regulation of Fe homeostasis required for photosynthesis. The comparison between Chlamydomonas and land plants is especially of interest as both are eukaryotes with nuclear control of regulation. However, in Chlamydomonas, acclimation to Fe deficiency will differ drastically from plants because the unicellular algal cell needs to only allocate Fe between different cellular compartments, and not across different tissues. 

Today, Fe is the fourth most abundant element in the earth’s crust [23] but is not readily bioavailable. Fe availability in soil depends on many factors, including pH and availability of other elements, but in general, soil Fe is predominantly found in Fe oxide, which is poorly bioavailable for plants [24]. In aqueous environments, dissolved Fe is more abundant in freshwater systems than marine systems [25]. In marine environments, dissolved Fe is one of the main limiting factors for photosynthetic output in areas of high nitrogen and phosphorous [26]. Land plants are found in varying soil habitats. *Chlamydomonas reinhardtii* is found in temperate soil environments [27]. Different Cyanobacterial strains are found in almost all environments including marine, freshwater, and terrestrial habitats [28]. While the Fe availability of these organisms’ natural environments may influence their responses to Fe limitation, most studies on regulation of Fe homeostasis are done in artificial environments. Chlamydomonas and Cyanobacteria are typically grown in agar or liquid culture, and plants are grown on agar or hydroponic conditions where few factors, other than Fe, are limiting. For plants on soil in laboratory settings, Fe availability can be decreased by addition of lime, which raises pH, while Fe chelates can be added to increase Fe absorption [29].

Here, we will review mechanisms of acclimation to Fe deficiency across green lineages, by comparing Fe metabolism of chloroplasts in land plants and in Chlamydomonas with Cyanobacteria.

## 2. Chloroplast Fe Use

The majority of chloroplast proteins are encoded in the nucleus, translated on cytoplasmic 80S ribosomes and imported into the organelle before maturation and assembly [30]. The chloroplast genome encodes a set of proteins that function in photosynthesis or chloroplast gene expression [31]. Both plant development and the environment affect chloroplast function, and therefore the expression and maturation of plastid-encoded and nucleus-encoded chloroplast proteins must be coordinated to respond to developmental and environmental cues [30]. Micronutrient availability (including Fe) is one important environmental variable. Due to its very low bioavailability, and the high photosynthetic requirement [7], Fe is one of the main nutrients limiting plant productivity.

Fe is required for biological processes because of its role as a protein cofactor. Fe cofactors exist in three main forms (heme, nonheme, and Fe–S clusters) to allow proteins to carry out functions such as catalysis, electron transport, and ROS-scavenging [10]. Fe is the most common metal cofactor and Fe cofactors provide a range of redox potentials for different protein functions [10]. The photosynthetic electron transport chain requires all three forms of Fe cofactors. The highest demand is for Fe–S clusters, with Photosystem I (PSI) subunits requiring three 4Fe-4S clusters, each Rieske subunit of the Cytochrome-*b_6_f* (Cyt-*b_6_f*) complex requiring a 2Fe-2S cluster and, Ferredoxin (Fd) requiring a 2Fe-2S cluster [32,33,34]. The Cyt-*b_6_f* complex also contains multiple heme cofactors for electron transport and exists as a dimer, for a total of 12 Fe atoms spanning the subunits [7]. Photosystem II (PSII) requires one nonheme Fe cofactor, but, unlike Fe in the rest of the photosynthetic electron transport chain, it is unlikely that this cofactor is involved in electron transport [35]. PSII also contains a cytochrome heme cofactor that has a photoprotective role [7].

### Fe Cofactor Assembly in Plastids

**Figure 2:**
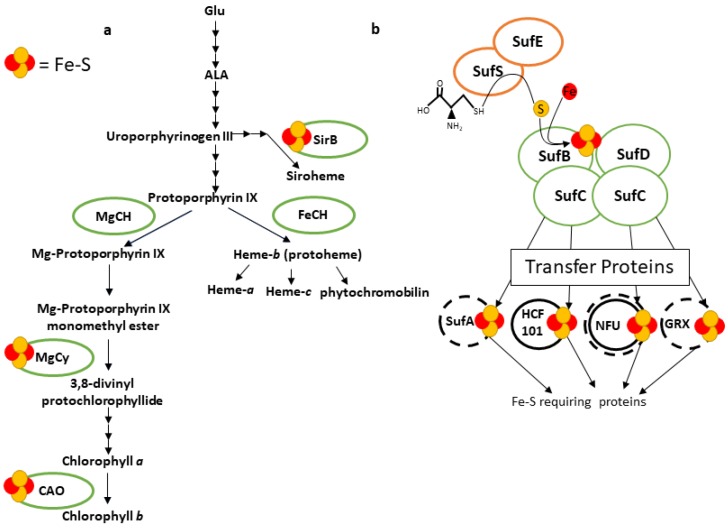
Biosynthesis of Fe cofactors in the chloroplast. (**a**) Tetrapyrrole biosynthesis. Tetrapyrrole biosynthesis produces heme, siroheme, and chlorophyll. Enzymes requiring Fe–S clusters are denoted. Tetrapyrrole biosynthesis begins with Glutamate (Glu) which is used to form aminolevulinic acid (ALA) which is converted into protoporphyrin IX (PPIX). The pathway then splits to either produce heme by insertion of Fe by Ferrochelatase (FeCH) or chlorophyll a and b by insertion of Mg by Magnesium Chlelatase (MgCH). Chlorophyll biosynthesis is catalyzed, in part, by the enzymes, Mg Proto IX Monomethyl Ester Cyclase (MgCy) and Chlorophyllide A Oxygenase (CAO) which require Fe–S clusters. Siroheme cofactor production branches before protoporphyrin IX is produced and is catalyzed by Sirohydrochlorin Ferrochelatase (SirB). Each arrow signifies one enzymatic step. (**b**) Sulfur Utilization Factor (SUF) Fe–S assembly. Fe–S assembly begins with cysteine desulferase via SUFS and SUFE. The Fe–S cluster is produced on the SUFBCD scaffold and then may be transferred to candidate carrier molecules, including SUFA, High Chlorophyll Fluorescence 101 (HCF101), Nitrogen Fixation U-Like (NFU), and monothiol glutaredoxins (GRX), for delivery to target proteins. Enzymes necessary for cysteine desulfurase are orange, enzymes of the major scaffold are green, and transfer proteins are black. Dashed lines for the carrier proteins indicate biochemical evidence of their role. Solid lines indicate genetic evidence of their role.

Relatively little is known about the maturation of nonheme Fe proteins in plants. In contrast, the synthesis and assembly of heme and Fe–S clusters is understood in greater detail. In plants, the synthesis pathway of heme and siroheme is localized in plastids. Siroheme, heme, and chlorophyll synthesis all branch off from the plastid tetrapyrrole pathway (Figure 2a) [36,37,38]. The tetrapyrrole pathway begins with three enzymatic steps whereby glutamate is used to form aminolevulinic acid (ALA), the tetrapyrrole precursor [38]. ALA is proposed to be maintained in two separate pools for heme and chlorophyll biosynthesis [39] and heme synthesis is directly linked to the amount of ALA present [40]. Eight molecules of ALA are used to form uroporphyrinogen III, which has the basic tetrapyrrole-conjugated ring structure. The pathway branches at uroporphyrinogen III to form on one hand siroheme, which requires the 2Fe-2S enzyme, Sirohydrochlorin Ferrochelatase B (SirB) [41], or on the other hand protoporphyrin IX (PPIX), the common precursor for chlorophyll and heme production [38]. Fe insertion into PPIX by Ferrochelatase leads to heme formation while Mg-ion insertion leads to functional chlorophyll [36]. High Chlorophyll Fluorescence 164 (HCF164/CCS5), a thioredoxin, and Cytochrome-c Deficient A (CCDA), a thylakoid thiol disulfide transporter, are proteins that are required for the correct insertion of heme into plastid cytochromes [42,43]. It is notable that several enzymes of heme and chlorophyll metabolism are Fe–S-cluster-dependent enzymes (Figure 2a).

Fundamental mechanisms of Fe–S cluster synthesis were first uncovered in nitrogen-fixing bacteria that utilize the Nitrogen Fixation (*nif*) operon [44,45]. It became apparent that housekeeping Fe–S proteins in bacteria requires another operon called Iron Sulfur Cluster (*isc*) [45], while a third bacterial Fe–S system—functioning under oxidative or low sulfur stress conditions—is encoded in the Sulfur Utilization Factor (*suf*) operon [46,47]. Fe–S cluster assembly by either the *nif/isc* or *suf* systems can be divided into three steps: mobilization of S from cysteine (Cys) by a Cys desulfurase, assembly of S with Fe on scaffolds to form an Fe–S cluster, and transfer to and insertion into apoproteins, for reviews see [48,49,50]. The first discovered Cys desulfurase was NifS (in the *nif* cluster), and later IscS and SufS were found to have the same function in the *isc* and *suf* operons, for a review see [51]. 

Homologues of the bacterial *nif*, *isc* and *suf* genes have been discovered in other organisms including yeast and plants for reviews see [50,52]. In plants there are two major Fe–S cluster biosynthesis machineries, one in mitochondria and one in plastids [52]. However, in analogy to other eukaryotes [50], the mitochondrial machinery is linked with a cytosolic Fe–S assembly system [53,54,55]. The ATP-binding cassette (ABC) transporter called ABC Transporter of the Mitochondrion 3, (ATM3) is required for cytosolic Fe–S assembly and likely exports glutathione disulfide that is thought to be used to provide S for cytosolic Fe–S assembly [55]. The mitochondrial machinery is homologous to that encoded by the bacterial *isc* operons, while several of the genes encoding the plastid machinery resemble bacterial *suf* operons (Figure 2b) [52,56]. 

Evidence for a plastid Fe–S cluster assembly system was obtained when Fd maturation was observed after in vitro import into isolated chloroplasts, which have their own Fe–S biosynthetic machinery [57,58]. Fe–S cluster assembly for Fd in isolated chloroplasts used cysteine as the sulfur donor and further required light or ATP and NADPH [59,60,61]. However, the Fe source for plastid Fe–S cofactor formation is unclear [62]. 

CpNifS/SUFS (called SUFS from hereon) is the only plastid stroma protein with Cys-desulfurase activity [63,64]. Arabidopsis SUFS is constitutively expressed in all major plant tissues at about equal levels [63]. Stromal fractions can mediate Fe–S cluster insertion into apoferredoxin in vitro, but this activity was lost when SUFS was depleted [65]. Inducible RNAi knockdown mutants of SUFS led to a gradual loss of all plastidic Fe–S proteins, causing chlorosis, thylakoid degradation, severe impairment of photosynthesis, and eventually death; thus, SUFS is required for all Fe–S formation in plastids [66]. SUFS is a type-II Cys-desulfurase, which in bacteria is activated by SufE. Plants have three nuclear encoded, plastid-targeted SufE-like proteins. SUFE1 forms a complex with SUFS in vitro and stimulates Cys-desulfurase activity 40–60-fold [67]. Homozygous *sufe1* knockout mutants are not viable [68]. Next to a SUFE-domain found in both prokaryotes and eukaryotes, the mature SUFE1 protein in plants has a BolA-like domain that is unique to higher plants and that may play a role in regulation via interaction with monothiol glutaredoxins, perhaps in response to redox status [69]. There are two additional SUFE proteins in plants: SUFE2 functions in Fe–S assembly in pollen and SUFE3 plays a role in plastid NAD synthesis and carries a 4Fe-4S cluster required for this activity [70]. 

In the bacterial *suf* system a complex of SufB, C and D forms an Fe–S assembly scaffold with ATPase activity [56]. Homologues of SufB, C and D are also present in plastids where they form a complex [71,72,73,74] (Figure 2b). Arabidopsis SUFB, SUFC and SUFD are known to be essential proteins, and inducible RNAi knockdown mutants of the *sufbcd* scaffold proteins had lower accumulation of Fe–S-requiring photosynthetic proteins, suggesting that the SUFBCD complex is required for the synthesis of all photosynthetic Fe–S clusters [74]. Other components of the plastid Fe–S machinery are the potential transfer proteins that serve to insert Fe–S clusters into Fe–S-requiring proteins [75]. Potential transfer proteins include Nitrogen Fixation U-Like 1-3 (NFU1-3) [64,76], CpIscA/SUFA, hereon called SUFA [77], and High Chlorophyll Fluorescence 101 (HCF101) [78,79]. Mutational loss of NFU2 or HCF101 results in defects in the maturation of specific subsets of Fe–S cluster proteins [76,79,80,81]. However, for a *sufa* loss-of-function mutant, no phenotype was described in mutant plants grown on regular soil [77,82]. Furthermore, two monothiol glutaredoxin (GRX) proteins (GRXS14/GRXS16) could serve as Fe–S scaffolds or transfer proteins, based on in vitro studies, but no strong phenotypes for loss of function mutants have been reported [83]. Finally, a plastid and mitochondrial dual localized protein called AtNEET, named for the NEET amino acid motif, has the capacity to bind an Fe–S center and it was proposed that AtNEET may participate in Fe–S export from the chloroplast [84]. A dominant negative mutation in AtNEET-affected chloroplast biogenesis, ROS homeostasis and plant Fe metabolism, but did not seem to affect expression of the Cyt-*b_6_f* Rieske protein [84,85]. 

## 3. Chloroplast Fe Transport and Storage

In photosynthetic plant cells, chloroplast Fe uptake systems are induced in the light, suggesting a link between Fe requirement and photosynthetic capacity [86,87,88]. Fe uptake in isolated pea chloroplasts was reported to be dependent on the pH gradient across the inner membrane which is maintained in the light [89]. Further, in mature chloroplasts, Fe homeostasis has been closely tied to circadian rhythm [90] and light–dark cycles through the Time for Coffee clock regulator (TIC) [91]. Circadian rhythms in etiolated seedlings (which have undeveloped, non-photosynthetic, chloroplasts) were unresponsive to low Fe, suggesting that as the chloroplast develops as a sink for Fe, a signal is produced to modulate the circadian clock based on Fe status [90]. Here we will briefly discuss Fe transport systems. For comprehensive reviews on Fe transport, see [92,93]. 

### 3.1. Chloroplast Fe Uptake 

**Figure 3:**
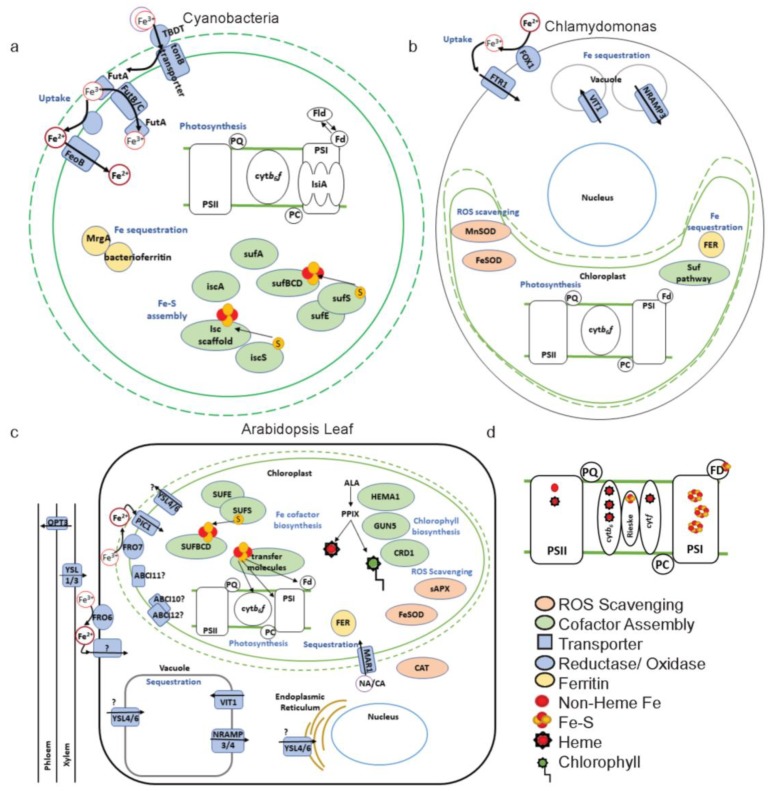
Fe-responsive proteins with relevance for photosynthesis in Cyanobacteria, Chlamydomonas, and land plants. Proteins that are discussed in this review for each species are presented (**a**) Fe-responsive proteins in Cyanobacteria, relatives to the ancestors of chloroplasts. On the outer membrane, Fe^3+^ is chelated by a siderophore and taken up through a TonB Dependent Transporter (TBDT). TonB spans the periplasmic space and the inner membrane to facilitate uptake through the TBDT. On the inner membrane, Fe is taken up as Fe^3+^ via the Fe Uptake Transporter, FutABC, system. Fe can also be taken up as Fe^2+^ after reduction, through Ferrous Iron Transporter B (FeoB). Fe is required for the photosynthetic electron transport chain. During Fe deficiency, Iron Stress Induced protein A (IsiA) can protect Photosystem I (PSI) and Ferredoxin (Fd) can be replaced by the non-Fe-requiring Flavodoxin (Fld) in several species. Two operons exist for Fe–S cluster assembly: Iron Sulfur Cluster (*isc*) and Sulfur Utilization Factor (*suf*). Fe is sequestered by ferritin molecules: bacterioferritin and MrgA. (**b**) Fe-responsive proteins in *Chlamydomonas reinhardtii.* Chlamydomonas takes up Fe^3+^ by a ferric iron permease yeast homologue (FTR1) after Fe^2+^ is oxidized by Ferric Oxidase 1 (FOX1). Fe is sequestered in the vacuole via Vacuolar Iron Transporter 1 (VIT1) and can be exported from vacuoles via Natural Resistance Associated Macrophage-like Protein 3 (NRAMP3). Chloroplast Fe is sequestered by Ferritin (FER). The ROS-scavenging SuperOxide Dismutases (SOD), FeSOD and MnSOD, are regulated in response to Fe deficiency. (**c**) Fe-responsive proteins in the leaf mesophyll cell with a focus on chloroplast proteins. From the xylem, Fe can be loaded into the phloem by Oligo Peptide Transporter 3 (OPT3) or to the mesophyll cell. For import into the mesophyll cell, Fe is exported from the xylem by Yellow-Stripe Like 1/3 (YSL1/3) presumably in a Fe^3+^-nicocianamine (NA) complex. Fe^3+^ is reduced at the leaf plasma membrane by Ferric Reductase Oxidase 6 (FRO6) and Fe^2+^ is taken up into the cell. Fe is reduced at the chloroplast envelope by FRO7 and taken up into the stroma by Permease in Chloroplasts 1 (PIC1). ATP Binding Cassette (ABC) proteins, ABCI11, ABCI10, and ABCI12 may also take up Fe into the chloroplast. YSL4/6 is proposed to be a chloroplast Fe exporter. Multiple Antibiotic Resistance1 (MAR1) may transport NA or citrate (CA) into the chloroplast to sequester free Fe. FER is also required to sequester Fe. Fe–S clusters are formed by the SUF pathway in the chloroplast and transfer molecules insert these Fe–S clusters into photosynthetic proteins. Heme and chlorophyll are produced in the tetrapyrrole pathway. Many enzymes for chlorophyll and heme production are Fe-responsive, including Genome Uncoupled 5 (GUN5), Glutamyl-tRNA reductase 1 (HEMA1), and Copper Response Defect 1 (CRD1). Within the chloroplast, during Fe deficiency, ROS-scavenging molecules, Stromal Ascorbate Peroxidase (sAPX) and FeSOD are downregulated in Fe deficiency. Catalase (CAT) is maintained or slightly downregulated during Fe deficiency. Fe is sequestered in the vacuole, where it is imported by VIT1 and exported by NRAMP3/4. YSL4/6 may also be a vacuolar Fe exporter. (**d**) Fe requirement of photosynthetic electron transport chain proteins. Symbols are explained in the legend.

Because Cyanobacteria are related to the evolutionary ancestors of chloroplasts, Fe uptake mechanisms in Cyanobacteria may be related to chloroplast Fe uptake. Both the chloroplast and Cyanobacteria contain an outer and inner membrane that Fe must cross for import [92]. In photosynthetic organisms several mechanisms can be used and combined for extracellular Fe uptake. In one mechanism, the organism produces siderophores, Fe-chelating molecules, which are secreted extracellularly to chelate Fe, and then the siderophore–Fe complex is taken up by the organism. In a second mechanism, Fe is reduced from Fe^3+^ to Fe^2+^ by a membrane-bound reductase enzyme, and then is taken up by a transmembrane Fe uptake protein. Cyanobacteria employ both strategies to import Fe across the outer and inner membranes (Figure 3a) [94,95,96]. On the outer membrane, Cyanobacterial species are thought to take up Fe into the periplasmic space through TonB Dependent Transporters (TBDT) [97]. *Anabaena* PCC 7120 secretes a siderophore and takes up the Fe siderophore complex by using a TBDT [98,99]. A *Synechocystis* PCC 6803 TBDT quadruple mutant had decreased Fe uptake rates compared to wild type [100]. Chloroplast outer membrane Fe specific transport proteins have not been characterized [92] but the relatively large pore size of outer membrane porins [101] may allow diffusion of Fe-chelator complexes to reach the inner membrane surface. In the periplasm, *Synechocystis* PCC 6803 binds Fe^3+^ by FutA of the Fe Uptake Transport system, FutABC. There is convincing evidence that reduction of FutA bound Fe^3+^ results in Fe^2+^ release from FutA, which is then transported through the inner membrane by the Fe uptake protein Ferrous Iron Transporter B (FeoB) [96]. However, there is also evidence for non-reduction-based uptake of Fe^3+^ by the FutABC uptake system in *Synechocystis* PCC 6803, in which Fe^3+^ is released from one molecule of FutA in the periplasm, and is transported through the FutB transmembrane protein where it is bound to a second molecule of FutA in the Cyanobacterial cytosol [96]. 

In higher plants, at both the plasma membrane and chloroplast membranes, Fe is reduced before uptake (Figure 3c). Arabidopsis Ferric Reduction Oxidase 6 (FRO6) functions to reduce Fe for import into the leaf cell and FRO7 reduces Fe for import from the cytoplasm into the chloroplast [29]. Expression of both FRO6 and FRO7 is induced during differentiation of leaf cells as chloroplasts mature [87]. Additionally, FRO6 expression is driven by light-responsive elements in its promoter [87]. FRO7 is especially linked with photosynthetic need, as Arabidopsis knockout mutants exhibited stunted growth, low electron transport, and decreases in the accumulation of Cyt-*b_6_f*, when grown on agar without sucrose [29]. However, increasing Fe supply to *fro7* could recover this phenotype, suggesting that there is either an alternative chloroplast reductase enzyme, or an alternative, currently unknown, Fe uptake pathway that does not require reduction. 

While the chloroplast seems to employ FRO7 to acquire Fe, it is unclear if the reductase activity for Fe is tied to a specific Fe transporter or if Fe reduced via FRO7 can be taken up by multiple Fe importers. Likely, there are multiple high- and low-affinity transport systems to modulate Fe movement into or out of the chloroplast based on chloroplast Fe need [102]. The most viable candidate chloroplast Fe importer is the inner-membrane-localized Permease In Chloroplast 1 (PIC1) (Figure 3c). *PIC1* and its homologue, *sll1656* from *Synechocystis* PCC 6803, complemented a yeast Fe uptake deficient mutant, suggesting a role in Fe import for both transporters [103]. Arabidopsis *pic1* mutants had severely underdeveloped chloroplasts along with an increased expression of the stromal Fe storage molecule, ferritin (FER), suggesting an increased capacity to store Fe. FER expression is presumably induced by ROS accumulation as a consequence of decreased accumulation of photosynthetic proteins [103]. PIC1 was originally characterized as a subunit of the chloroplast protein importer (TIC21) [104], but all expressed chloroplast proteins in *pic1* were processed to their mature form, indicating that they were imported into the chloroplast [103]. Therefore, Duy et al. [105] propose that PIC1 may closely associate with a TIC protein on the inner chloroplast membrane to coordinate Fe import and Fe cofactor assembly with protein maturation. 

A second candidate transfer system that may mediate chloroplast Fe uptake is comprised of the ATP-Binding Cassette proteins, ABCI10, ABCI11/NAP14, and ABCI12. Arabidopsis ABCI10 and ABCI11/NAP14 GFP fusion proteins were localized to the chloroplast, and most likely to the inner envelope [106,107]. Both ABCI10 and ABCI11/NAP14 knockout mutants share the severely undeveloped chloroplast phenotype of PIC1 and also exhibit decreases in FRO7, and PIC1 transcripts [107]. ABCI10 is predicted to interact with ABCI12, a transmembrane protein localized to the chloroplast inner membrane and to provide ATPase activity for ABCI12. However, the interaction of ABCI10 and ABCI12 is only based on colocalization in fluorescence microscopy for ABCI10:GFP and ABCI12:YFP, and ATPase activity of ABCI10 has not been directly measured [107]. While ABCI11/NAP14 may be important for chloroplast Fe, many questions remain about its involvement in transport, for instance the direction of ABCI11/NAP14-mediated transport is unknown [106]—i.e., it could be used with an Fe import system, an Fe export system or not involved in Fe uptake.

### 3.2. Chloroplast Fe Export 

Fe export from the chloroplast is equally important as Fe uptake both to ensure that the chloroplast is not overloaded with Fe, which can lead to production of free radicals by Fenton reactions, and to export Fe from the chloroplast during senescence. Export from chloroplasts in *Arabidopsis* may be mediated by two Yellow Stripe Like proteins (YSL4 and YSL6). The double mutant, *ysl4/6* over accumulated Fe in the chloroplast and induced FER expression [108]. However, localization of YSL4/6 has been a matter of debate based on the method used. In immunolocalization experiments, fluorescently tagged antibodies for YSL4/6 were localized to the chloroplast [108], while YSL4/6-GFP fusion proteins localized to the tonoplast and the endoplasmic reticulum (Figure 3c) [109]. Both a proposed function as a chloroplast Fe exporter or as a tonoplast Fe importer could explain the accumulation of Fe in the chloroplast. Clearly, the loss of a chloroplast Fe exporter would result in higher levels of Fe remaining in the chloroplast. However, the loss of a tonoplast Fe importer could also lead to increases in chloroplast Fe, as less Fe can be distributed into the tonoplast it may be sequestered instead by FER in the chloroplast. Regardless of the location of YSL4/6, it does appear to have a role in modulating intercellular Fe. However, the ability of YSL4/6 to transport Fe has not been directly measured and the sensitivity to Fe of *ysl4/6* seems to be dependent on the growth conditions; thus it was suggested that YSL4/6 may transport manganese or nickel instead of Fe [109]. 

### 3.3. Fe Sequestration 

Fe is thought to be transported while bound to Fe chelators, such as nicotianamine (NA) and citrate. *Brassica napus* chloroplasts, had a higher rate of Fe uptake when Fe was supplied as Fe(III)-citrate compared to when it was supplied as Fe(III)- NA or Fe(II)-NA [110]. Once inside the chloroplast, the majority of the Fe is found as heme or Fe–S clusters [111]. A possible chelate transporter, Arabidopsis Multiple Antibiotic Resistance 1 (MAR1) has been localized to the chloroplast inner envelope. MAR1 was discovered as a chloroplast importer of antibiotics but more likely evolved to function in Fe uptake, as its expression has been linked to Fe status [112]. Most likely, MAR1 functions to couple import of Fe chelators with that of Fe so that once inside the chloroplast (Figure 3c), Fe is chelated to avoid Fenton reactions. However, the specific chelator that may be imported by MAR1 is unknown. 

**Figure 4:**
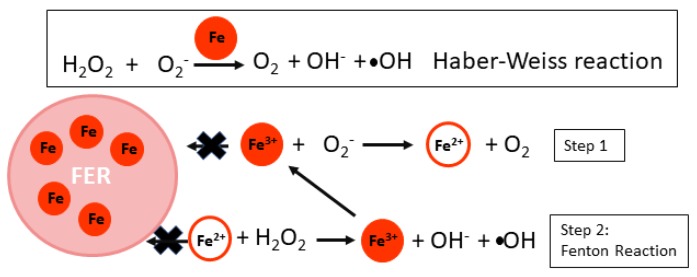
Role of Ferritin (FER) in preventing Haber–Weiss and Fenton reactions that can perpetuate the production of reactive oxygen species (ROS). FER is expressed under normal conditions to prevent accumulation of free Fe and avoid excessive build up of ROS. If FER cannot capture free Fe, Haber–Weiss reactions, which are catalyzed by free Fe, can lead to the build up of hydroxyl radicals (•OH). These reactions occur in two steps. First, Fe^2+^ and oxygen are produced from Fe^3+^ and superoxide. Second, the Fe^2+^ can react with hydrogen peroxide (H2O2) to produce Fe^3+^ and harmful hydroxyl radicals in a Fenton reaction.

Ferritin (FER) is the major Fe storage molecule located in the chloroplast and is vital to modulating the amount of free Fe in the chloroplast for use (Figure 3c) [113]. *Arabidopsis* has four ferritin genes: FER1, FER3, and FER4 are major leaf Ferritins, while FER2 is expressed in the seed [114]. Ferritins are found in bacteria and animals as well as across green lineages [115]. Ferritin proteins complexes make a shell-like structure that holds Fe^3+^ bound to organic phosphate [116]. One Ferritin shell is composed of 24 FER molecules [116] and ferritin from legumes was found to hold at least 1000 Fe atoms [117]. The major function of FER in plastids is to scavenge free Fe to eliminate the threat of ROS production (Figure 4) [113]. Free Fe in the presence of oxygen can lead to the accumulation of harmful hydroxyl radicals (•OH) and hydrogen peroxide (H2O2) via Fenton and Haber–Weiss reactions, which disrupt membrane integrity and protein structure (Figure 4) [118]. FER is especially important for chloroplast protection during Fe toxicity, where its expression is stabilized to strengthen Fe storage. FER triple mutants (*fer1/fer3/fer4*) had limited growth and enhanced catalase and ascorbate peroxidase activity at high levels of Fe, suggesting an increase in ROS protection mechanisms [113]. Cyanobacterial bacterioferritin is required for buffering Fe stores and a second class of FER (MrgA) is suggested to provide redundancy for bacterioferrtins in Fe deficiency to avoid oxidative damage [119], which may be a consequence of Fe deficiency depending on the severity of Fe limitation. The function of FER in Fe homeostasis will be further discussed in the following sections.

## 4. Acclimation to Low Fe

During Fe deficiency a photosynthetic organism must acclimate both locally and systemically to alter growth and metabolism. The systemic response to low Fe becomes more complex as the number of cells and structures in the organism increases. For example, unicellular Cyanobacteria will have one overall response as the organism will sense Fe and respond in the single cell. Filamentous Cyanobacteria, such as *Anabaena variabilis*, that have specialized cells for different metabolic functions, such as nitrogen fixation, may sense Fe locally in photosynthetic cells but will have to respond systemically to acclimate other cells to the deficiency in photosynthetic output [120]. In contrast, the unicellular eukaryotic alga, Chlamydomonas will have local responses in different cellular compartments, and these responses will need to be coordinated by signaling between organelles and nucleus [121]. In land plants, not only do responses need to be coordinated in each cell, but also between different organs. For instance, plants induce root Fe uptake based on shoot Fe needs. Therefore, in land plants, there are distinct but coordinated local responses to Fe deficiency in leaves, vasculature, roots, and reproductive structures [122,123]. 

In green organisms, photosynthesis is a major target for regulation under low Fe availability. During Fe deficiency, photosynthetic organisms become chlorotic as they decrease activity of the chlorophyll biosynthesis pathway [124]. Chlorophyll biosynthesis requires Fe for Chlorophyllide A Oxygenase (CAO) and the Copper Response Defect 1 (CRD1/CHL27) subunit of Mg Proto IX Monomethyl Ester Cyclase (MgCY) [38]. In plants, chlorosis begins in the young leaves with developing chloroplasts, reflecting an inability to remobilize Fe from mature tissue to developing leaves. Photosynthetic electron transport activity when measured by chlorophyll fluorescence is specifically inhibited downstream of PSII as indicated by a lower φPSII parameter (indicating electron transport downstream of PSII) that is decreased to a stronger extent than maximum efficiency of PSII (F_v_/F_m_) [125,126]. Comparatively, cellular respiration rates are much less affected in a mild Fe deficiency in *Arabidopsis* [126], suggesting that chloroplast metabolism, as opposed to that of the mitochondria, is a major target of the Fe deficiency response. 

The regulated response to Fe deficiency depends greatly on the severity of the iron deficiency and developmental stage of the organism. In Chlamydomonas and Arabidopsis, severity of Fe deficiency can be determined by both the length of Fe deficiency and the fold change in available Fe [121,125,127]. A more severe Fe deficiency can lead to irreversible damage to photosynthetic electron transport chain and secondary stress responses [121]. Symptoms of less severe Fe deficiency in Arabidopsis can be recovered by resupplying plants with sufficient levels of Fe [126]. A list of Arabidopsis chloroplast homeostasis proteins and their orthologues in Cyanobacteria and Chlamydomonas are listed in Table 1. 

### 4.1. Increase Fe Uptake

The increase in Fe uptake in response to Fe deficiency in photosynthetic organisms has been well documented and upregulation of uptake systems are common markers of Fe deficiency [16,17,131,132,133]. Cyanobacteria are known to increase Fe uptake by upregulating FutC and FeoB (Figure 3a) [16,17]. Chlamydomonas employs an oxidation strategy for Fe uptake, similar to yeast, where Fe^2+^ is oxidized by a multicopper ferroxidase (FOX1) and then taken up by a ferric Fe permease yeast homologue (FTR1) [15]. Chlamydomonas increases Fe^3+^ uptake by upregulation of FOX1 and FTR1 expression in response to deficiency (Figure 3b) [15,133]. 

In plants, Fe uptake at the root has been thoroughly studied [21,123,134]. In short, the dicot plant, *Arabidopsis*, acidifies the rhizosphere by the proton pump, H+ATPase 2 (AHA2); reduces the poorly bioavailable Fe^3+^ to Fe^2+^ via the Fe Reductase Oxidase 2 (FRO2) enzyme; and finally, takes up Fe^2+^ from the soil through Iron Regulated Transporter 1 (IRT1) [21,134]. 

### 4.2. Metabolic Remodeling 

Plants remodel metabolism locally, in both the shoots and roots to respond to Fe deficiency. In the roots, C metabolism, ROS metabolism and N metabolism have all been found to be targets of metabolic remodeling in response to Fe deficiency [122,135]. Leaf metabolic remodeling coordinates downregulation of photosynthetic output with photoprotective mechanisms [124]. Early in the Fe deficiency response, gene expression of mRNAs that encode enzymes in the tetrapyrrole pathway are downregulated. The targeted mRNAs include *HEMA1* which encodes for an enzyme needed for ALA synthesis in the tetrapyrrole pathway, and a geranylgeranyl reductase (*CHLP*), a phytochlorophyllide oxidoreductase (*PORB*), and Genome Uncoupled 5 *(GUN5*) which are all required for chlorophyll production (Figure 3c) [124]. In addition, N metabolism has been found to be altered in leaves, with decreased expression of nitrate reductase, which requires Fe, and altered amino acid accumulation [136]. Chlorophyll and chloroplast protein biosynthesis, both require N assimilation [137], and therefore, the alterations to N metabolism could represent adjustments these pathways during Fe deficiency. Additionally, mRNA encoding the Fe-responsive protein, Conserved in Green Lineage and Diatoms 27 (CGLD27) is upregulated and this is predicted to function in quenching of ROS that results from photooxidative damage [124]. Other ROS-scavenging molecules in the plastid, such as the Fe-requiring stromal ascorbate peroxidase (sAPX), are downregulated, at least at the transcriptional level, during Fe deficiency [126], suggesting that specific ROS reduction systems are employed for photoprotection. Overall, metabolic remodeling in response to Fe deficiency suggests the plant decreases chlorophyll synthesis and increases mechanisms to protect from ROS.

The metabolic remodeling on low Fe in Chlamydomonas depends on the growth requirements of the organism, as Chlamydomonas can grow both photoautotrophically and photoheterotrophically [121]. When grown photoautotrophically, carbon assimilation by means of photosynthesis is maintained on low Fe. When grown photoheterotrophically, in which cells are supplied with light for energy production and an organic carbon source, Fe deficiency results in a similar response as seen in plants with a maintenance of respiration and strong decreases to photosynthetic electron transport [121]. In photoheterotrophic conditions, the ascorbate concentration was reported to increase tenfold in the cell during Fe deficiency [138]. Chlamydomonas grown photoheterotrophically had increased xanthophyll cycle pigments but a decreased induction of Non-Photochemical Quenching (NPQ) during Fe deficiency, suggesting that a decreased rate of electron transport did not allow for acidification of the lumen needed for induction of NPQ [139]. In photoautotrophic conditions, Chlamydomonas maintained NPQ in response to Fe limitation [139]. These changes to metabolism in chloroplasts of Fe-deficient Chlamydomonas suggest a need to increase photoprotective mechanisms and defenses against ROS. 

### 4.3. Fe Utilization 

A second major acclimation to Fe deficiency in photosynthetic organisms is Fe economy, or changes in Fe utilization to prioritize specific Fe-requiring proteins and pathways over others when Fe-limited [121]. Fe economy is commonly studied by comparing proteomic and transcriptomic changes in response to Fe limitation. Photosynthetic organisms share overall similarities in Fe economy strategies and, specifically, photosynthesis, Fe–S cluster assembly, and Fe sequestration are targeted for downregulation (Figure 3) [122,124,126,140]. Prioritized pathways include respiration and ROS-scavenging [122,126,135]. Within each of these pathways, specific proteins are downregulated, upregulated, or maintained to produce the response to Fe deficiency, and these specific molecular responses are detailed in the remainder of this section. 

The downregulation of photosynthetic electron transport in response to Fe deficiency is conserved across green lineages. Interestingly, several Cyanobacteria, Chlamydomonas, and land plants all downregulate Ferredoxin (Fd) and the cytochrome-*b_6_f* complex (Cyt-*b_6_f*) proteins after Fe deficiency, presumably to economize Fe for use in other cellular functions (For iron need of photosynthetic proteins see Figure 3d) [122,124,126,135,139,141]. In Arabidopsis, downregulation of transcripts encoding these photosynthetic proteins is ordered to first downregulate Fd and then components of the Cyt-*b_6_f* complex [126]. Downregulation of Arabidopsis Fd2 is especially drastic. After a week of mild Fe deficiency in *Arabidopsis*, Fd accumulated to only 8% of its original levels [126]. 

Only in the most severe cases of Fe deficiency are Photosystem I and II (PSI and PSII) subunits downregulated in plants [124,126,127,142]. In plants, PSI is more affected by Fe deficiency than PSII. In Fe-deficient Arabidopsis seedlings, Rodriguez-Celma et al. [124] found strong downregulation of PSI subunits, and Light Harvesting Complexes (LHCs). A proteomics approach analyzing thylakoid membranes of six-week-old hydroponically grown Arabidopsis plants after one week of Fe deficiency found that accumulation of many LHC protein are decreased, along with a strong decrease in PSI subunits, PSAB, PSAC, and PSAD [127]. PSII subunits and chloroplast ATP synthase also decreased but, some subunits from these complexes increased in response to Fe deficiency including, PSBS, PSBT and PSBK [127]. Decreases in LHC protein abundance along with PSAC and PSAD were also observed in rice in response to severe Fe deficiency [13]. Both PSII and PSI decreased in Fe-deficient spinach thylakoids. The ratio of the main components that comprise each photosystem did not differ from that of control plants suggesting an overall decrease in the number of intact photosystems [143]. 

The response to downregulate PSI subunits late in the Fe deficiency response is conserved across green lineages. Cyanobacteria also downregulate PSI subunits in the Fe deficiency response [141]. In addition, the Fe deficiency response in Cyanobacteria PSI includes ultrastructure remodeling. *Synechococcus* PCC 7942 and *Synechocystis* PCC 6803, normally rely on a trimeric PSI, but Fe deficiency resulted in accumulation of PSI monomers which decreased electron flow [144]. PSI reduction is also not observed in Chlamydomonas until severe Fe starvation. During Fe limitation, mild changes to PSI function were observed but were attributed to decreased PSI association with LHCs [16]. In Chlamydomonas it was not until severe Fe starvation that PSI subunits were downregulated [121,145]. 

Chloroplast Fe–S assembly is a second major target of Fe deficiency. Regulation of the Suf Fe–S assembly in response to Fe limitation is dependent on the organism. In land plants, the SUFB subunit of the SUFBCD Fe–S assembly scaffold was found to be an early downregulated target of Fe deficiency [126,140,146]. While SUFB was the only plant SUF component to be downregulated at both the transcript and protein level, Fe–S transfer molecules of the SUF pathway (SUFA, NFU, and HCF101) decreased in protein accumulation while transcript levels were maintained [126,147]. Further, a loss of SufA and IscA components of the Cyanobacteria, *Synechococcus* PCC 7002, Fe–S assembly led to induction of Fe deficiency responses [148].

Perhaps unsurprisingly, Fe sequestration is generally downregulated in Fe deficiency to increase Fe availability. In plants, it is well known that the chloroplast Fe storage ferritin molecules (FER) are downregulated quickly after low Fe is induced [116,149,150]. Conversely, Chlamydomonas *FER* expression is induced in response to low Fe perhaps to link Fe status and oxidative stress responses [151,152]. Chlamydomonas FER knockdown mutants had slower PSI degradation and, therefore, FER may be important for sequestering free Fe as PSI is degraded in severe Fe deficiency [152]. While knockout mutants of FER in plants only presented a phenotype under Fe toxicity [113], knockout mutants of bacterioferritin in the Cyanobacterium *Synechocystis* PCC 6803 resulted in an Fe deficiency response under normal Fe conditions [129]. 

Fe sequestration is also regulated by increasing availability of vacuolar Fe during deficiency. In Arabidopsis, in response to Fe deficiency, Vacuolar Iron Transporter 1 (*VIT1*), responsible for vacuolar Fe loading, is downregulated, while Natural Resistance Associated Macrophage-like Protein 4 (*NRAMP4*), a vacuolar Fe exporter, is upregulated, suggesting less Fe is sequestered into the vacuole [149]. In Chlamydomonas, the *VIT1* homologue is upregulated during Fe deficiency and is predicted to sequester Fe released from the chloroplast, while *NRAMP4* is also upregulated [153]. The upregulation of both *VIT1* and *NRAMP4* in Chlamydomonas may allow the cell to control the amount of Fe in the cytosol. However, data is limited on the function of upregulating both a vacuolar Fe importer and exporter during Fe deficiency. The decrease in sequestration must be tightly regulated to avoid toxic side effects of free Fe. Thus, in Rice, Yellow Stripe Like 15 and 2, which are proposed to transport nicocianamine– or citrate–metal complexes across membranes were also upregulated in shoots in response to Fe deficiency, perhaps to bind free Fe released as Fe-requiring proteins are degraded [154]. 

The decreases in photosynthetic electron transport and Fe sequestration in response to low Fe supply may make the chloroplast more vulnerable to damage caused by ROS. In *Arabidopsis*, only 33% of mRNAs encoding ROS-scavenging proteins were regulated during low Fe, and of these, about half were downregulated [11]. Stromal Ascorbate Peroxidase (*sAPX*) and Fe Superoxide Dismutase (*FeSOD*) in the chloroplast were also downregulated during low Fe in Arabidopsis [126,135,150,155], while others, like catalase (CAT), were found slightly up or downregulated (Figure 3c) [126,135]. Interestingly, FeSOD is upregulated by low copper (Cu) availability [156]. A known consequence of Fe deficiency is an increase in uptake of Cu [20,150]. Thus, the decreased expression of FeSOD during Fe deficiency may be regulated indirectly, in response to increased levels of Cu. Conversely, Chlamydomonas FeSOD is maintained during Fe deficiency and it is possible that the required Fe cofactor for this protein is recycled from Fe released when Fe-requiring photosynthetic proteins are degraded [157]. Chlamydomonas also induced expression of MnSOD during low Fe, which is further regulated by free Fe and H2O2 (Figure 3b). MnSOD is otherwise not expressed and seems to be regulated by Fe status [157]. 

### 4.4. Compensatory Responses to Fe Deficiency

An interesting Fe deficiency response of some Cyanobacteria and some algae is the ability to replace or protect proteins that are affected by Fe deficiency (Figure 3a,b). Specifically, these organisms can replace Ferredoxins with Flavodoxin (Fld), a non-Fe-requiring protein when Fe-limited, which may allow them to maintain photosynthesis in Fe-limited conditions, reviewed in [158]. This compensatory mechanism has been lost in land plants and Chlamydomonas [159]. Interestingly, the insertion of bacterial flavodoxin into tobacco enhanced tolerance to Fe deficiency by preventing loss of photosynthetic components [160]. In addition, Cyanobacteria can protect PSI by expressing the protective protein, Iron Stress Induced Protein A (IsiA) (Figure 3a), during Fe deficiency. It is thought that IsiA associates with PSI to increase efficiency of electron transport to PSI to avoid oxidative damage [161].

## 5. Regulating Acclimation to Low Fe

Both local and systemic responses to Fe deficiency must be tightly regulated to coordinate Fe uptake with Fe needs during deficiency. To quickly adjust Fe homeostasis to changes in Fe status, regulation is transcriptional, post-transcriptional, and post-translational [123]. Regulatory mechanisms of responses to Fe deficiency in Cyanobacteria, Chlamydomonas, and land plants are largely specific to each organism. Further, in eukaryotic photosynthetic organisms, regulation of Fe deficiency is more complex, as the organism must coordinate Fe needs of organelles through retrograde signaling. The regulatory mechanisms to Fe deficiency of Cyanobacteria, Chlamydomonas and land plants are discussed in the following sections. 

### 5.1. Root Regulation of Fe Uptake 

In plants, the regulation of root Fe uptake has been extensively studied, and some of the Fe-responsive transcription factors identified in the root may regulate shoot responses to Fe limitation as well. At the root surface Fe is reduced by FRO2 and then taken up in the root symplast through IRT1. FRO2 and IRT1 expression is positively regulated by a cascade of Basic Helix Loop Helix (bHLH) transcription factors. Of the 133 bHLH transcription factors in *Arabidopsis*, 16 have a known role in Fe homeostasis [21,123,162,163]. Ethylene Response Factor (ERF) family transcription factors have also been associated with the root Fe uptake response [164,165]. The root specific bHLH FER-Like Fe Deficiency Induced Transcription Factor (FIT) is stabilized by two Ethylene Insensitive transcription factors, Ethylene Insensitive 3 and Ethylene Insensitive 3-Like 1(EIN3, EIL1) [166]. In terms of plastid regulation, ERF transcription factors may play a role in retrograde signaling in Fe deficiency [167]. The following sections will discuss possible regulation of plastid Fe deficiency responses.

### 5.2. Transcriptional Regulation 

**Figure 5:**
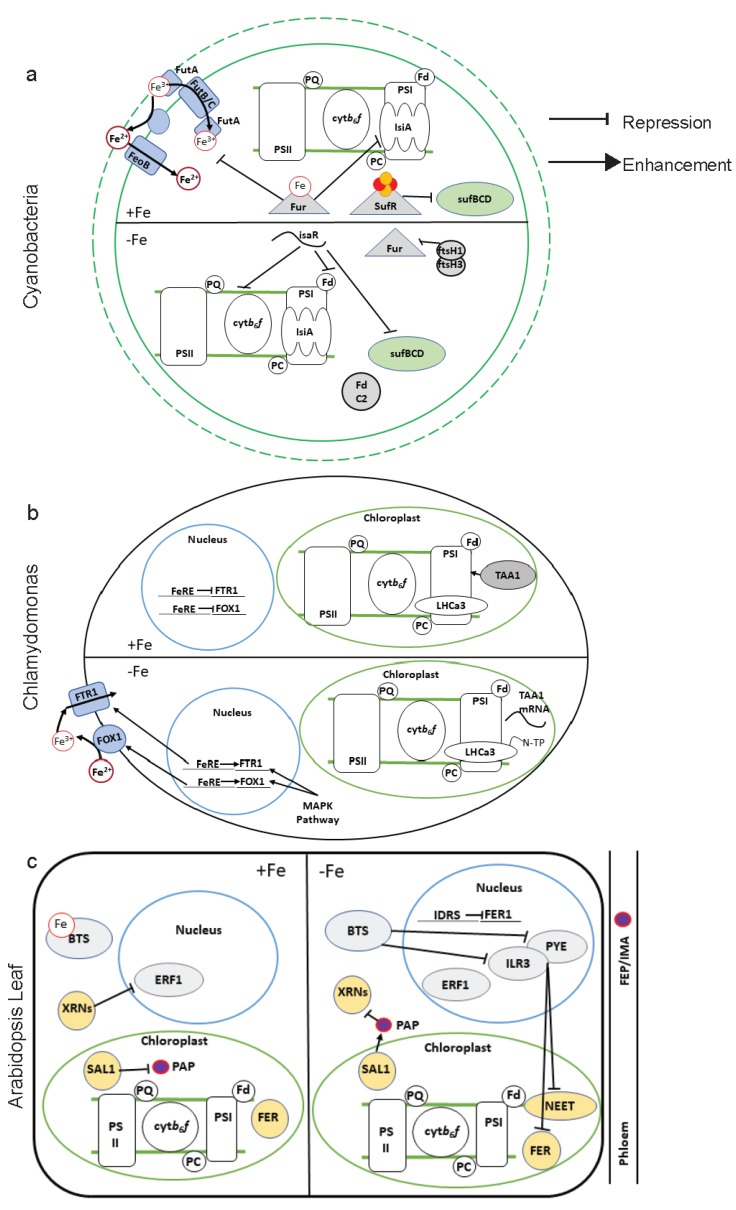
Regulation of Fe deficiency responses in photosynthetic organisms. (**a**) Regulation of Fe deficiency responses in Cyanobacteria. Fe Uptake Regulator (Fur) is the master regulator of Fe responses and acts as a transcriptional repressor under sufficient Fe. Fur represses the Fe uptake machinery as well as Iron Stress Induced protein A (IsiA). During Fe deficiency Fur is degraded by the protease complex, Filamentous H 1/3 (ftsH1/ftsH3). The expression of the Sulfur Utilization Factor (suf) operon is fine-tuned by the combined action of SufR and the small RNA called Iron Stress Activated RNA 1 (IsaR1). SufR protein, when bound to an Fe–S cluster, represses the *suf* scaffold to avoid excess Fe–S formation. IsaR1 is induced by Fe deficiency and represses transcripts for the sufBCD scaffold during Fe deficiency together with Cytochrome-*b_6_f* (Cyt-*b_6_f*) complex and Ferredoxin (Fd1). Ferredoxin C2 (FdC2) is hypothesized to regulate IsiA. (**b**) Regulation of Fe deficiency adjustment in Chlamydomonas. The Fe uptake machinery is regulated transcriptionally by Fe-responsive Elements in the promoters of the genes for the ferric iron permease (FTR1) and Ferric Oxidase 1 (FOX1) and by phosphorylation via the Mitogen Activated Protein Kinase (MAPK) pathway. Light Harvesting Complex A3 (Lhca3) undergoes N-terminal processing (N-TP) during Fe deficiency. Translation of PsaA1 (TAA1) stabilizes PSI under normal Fe conditions but the TAA1 protein is degraded during Fe deficiency, but TAA1 mRNA remains. (**c**) Regulation of Fe deficiency acclimation in the chloroplast. Fe deficiency responses are regulated by Basic Helix Loop Helix (bHLH) transcription factors, and possibly Ethylene Response Factor (ERF) transcription factors. The bHLH transcription factors, IAA-Leucine Resistant 3 (ILR3) and Popeye (PYE) interact to repress ferritin (FER) and NEET. The ubiquitin ligase, Brutus (BTS) negatively regulates bHLH transcription factors. Iron Dependent Regulatory Sequence (IDRS) elements in the promoter of Ferritin (FER1) mRNA repress FER expression under Fe deficiency. The PAP/SAL signaling pathway may influence responses to Fe deficiency. 3′-phosphoadenosine 5′-phosphate (PAP) is a signaling molecule that can move between cellular compartments and inhibit exonucleases (XRNs). SAL1 enzyme regulates levels of PAP. XRNs can degrade Ethylene Response Factor 1 (ERF1) mRNA resulting in less ERF1 protein accumulation. Targets of ERF1 in Fe deficiency have not presently been identified. During Fe deficiency, the Ironman/Fe Uptake Inducing Peptide (IMA/FEP) signaling peptides are transported in the phloem in order to signal upregulation of Fe uptake in the root.

In the Cyanobacteria strains, *Anabaena* PCC 7120 and *Synechococcus* PCC 7942, the regulation of Fe uptake is mediated by the transcription factor Ferric Uptake Regulator (Fur), reviewed in [168]. Fur can act both as a transcriptional repressor and activator but during Fe sufficiency binds Fe and acts to repress Fe-related gene expression (Figure 5a) [168].

Chlamydomonas regulation of Fe uptake is transcriptionally dependent on Fe-responsive Elements (FeRE) in the promoters of genes that encode key components of the Fe uptake machinery (FOX1, and FTR1; Figure 5b) [169,170]. The expression of the Fe uptake protein, FTR1, was both positively and negatively regulated by separate FeRE promoter elements [171]. Additionally, the Fe uptake machinery at the cell membrane is positively regulated transcriptionally via the Mitogen Activated Protein Kinase (MAPK) phosphorylation pathway, as RNAi knockdown mutants of this pathway had reduced mRNA levels of uptake machinery (Figure 5b) [172].

In plants, some of the bHLH transcription factors that are known to regulate root responses have also been found to be expressed in the shoots including bHLH104, Popeye (PYE), bHLH100/101, IAA-Leucine Resistant 3 (ILR3), and the recently identified, Upstream Regulator Of IRT1 (URI) (Figure 5c) [124,173,174,175,176]. Interestingly, in the leaf, an ILR3 overexpressor displayed repression of Cyt-*b_6_f* and PSI expression [177]. Additionally, ILR3 also interacts with PYE to negatively regulate expression of the chloroplast Fe storage molecule, ferritin [178,179]. Thus, ILR3 may be indirectly involved in initiating plastid transcriptional regulation of Fe homeostasis. 

Ethylene Response Factor (ERF) transcription factor regulation may also be involved in retrograde signaling in the leaf Fe deficiency response [167]. The PAP/SAL1 retrograde signaling pathway involves the metabolite, 3′-phosphoadenosine 5′-phosphate (PAP), which inhibits 5′-3′ exoribonucleases (XRNs), and the SAL1 enzyme that regulates PAP levels by dephosphorylation [180]. Knockout mutants of SAL1 and PAP targeted XRNs had higher accumulation of shoot and root Fe and higher expression of the FER molecules compared to WT [167]. Additionally, the *sal1* and *xrn* knockout mutants had increased expression of the ERF1 transcription factor mRNA, compared to WT, which is typically degraded by exoribonucleases 4 (XRN4) in the PAP/SAL1 pathway [167]. Thus, the ERF family may negatively regulate systemic Fe deficiency responses along with the PAP/SAL1 retrograde signaling pathway. 

Plant FER1 is regulated transcriptionally by an Iron Dependent Regulatory Sequence (IDRS) in the promoter, which is required for repression under Fe deficiency [116]. IDRS elements have been found in Arabidopsis and *Zea mays* FER1, and a similar sequence has been found in Soybean ferritin [116].

### 5.3. Post-Transcriptional Regulation 

The Cyanobacterial Fe-responsive fur transcription factor is regulated post-transcriptionally by small RNAs and the Filamentous H1/3 (ftsH1/ftsH3) protease complex [168]. Additionally, regulation of photosynthetic proteins in response to Fe deficiency in Cyanobacteria is mediated by a small RNA, Iron Stress Activated RNA 1, IsaR1 (Figure 5a) [141]. IsaR1 directly inhibits translation of photosynthetic proteins and prevents expression of SufBCD transcripts. 

In Chlamydomonas, the Translation of PsaA1 (TAA1) protein is required for efficient translation of PsaA [181]. TAA1 is thought to interact with the 5′UTR of PsaA mRNA to prevent degradation by 5′-3′ exonucleases under Fe sufficient conditions. PsaA mRNA and protein is absent in a TAA1 knockout mutant. During Fe limitation, TAA1 protein accumulation decreased, but TAA1 mRNA remained abundant, while PsaA protein and mRNA abundance decreased. This regulation of PsaA may allow for quick attenuation of PSI to Fe limitation.

### 5.4. Post-Translational Regulation 

In Chlamydomonas, in relation to photosynthetic regulation, key is the N-terminal processing of LHCa3, which has been found to stabilize LHC1 and protect the cell from loss of PSI function in Fe deficiency [182]. 

In plants, post-translational regulation of root and shoot Fe deficiency responses are regulated by E3 ubiquitin ligases, Brutus (BTS) and Brutus Like (BTSL) (Figure 5c) [183,184,185]. In *Arabidopsis*, turnover of ILR3, which is expressed in both roots and shoots, is thought to be regulated by BTS [183]. Thus, while translational regulation is required to ramp up the systemic Fe deficiency response, ubiquitin ligases are required for attenuating and coordinating the response. 

Recent evidence suggests that post-translational regulation by protein phosphorylation is necessary in the root Fe deficiency response in plants. Phosphorylation of the newly identified bHLH transcription factor Upstream Regulator of IRT1 (URI) was required to regulate bHLH038/039 and bHLH100/101 to upregulate the root uptake response, but not for downregulation of plastid localized Ferritin molecules and stromal Ascorbate Peroxidase (sAPX) [176]. Therefore, while phosphorylated URI may be vital in regulation of Fe uptake, it may not play a direct regulatory role in chloroplast Fe economy and metabolic remodeling. 

The chloroplast Fe–S cluster assembly machinery is integrally tied to regulation of photosynthesis in response to Fe deficiency. For instance, the response to downregulate SUFB of the SUF Fe–S cluster assembly may be one way in which photosynthetic electron transport proteins are post-translationally regulated since Fe–S clusters are required for photosynthetic proteins. In Arabidopsis RNAi inducible knockdown mutant lines of SUFB, all photosynthetic electron transport Fe–S proteins including Fd were lost [74]. The decrease in Fd seems specific to the loss of SUFBCD scaffold proteins as Fd was stable in Arabidopsis RNAi inducible knockdown mutant SUFS lines unless SUFS was knocked down during the seedling stage [66]. However, clearly the photosynthetic response to Fe deficiency is not solely regulated by loss of Fe–S clusters as photosystems are maintained until Fe deficiency becomes severe, and SUFB protein accumulation decreases early after Fe limitation [126]. Additionally, the putative Fe–S transfer protein, AtNEET, seems to regulate intracellular Fe. AtNEET dominant negative mutants over-accumulated Fe in the chloroplast, had chlorosis, and induced the root Fe uptake system [84] suggesting that intracellular Fe–S homeostasis is key in regulating both local and long-distance Fe deficiency responses. 

### 5.5. Fe Sensing

For regulation to occur, Fe status of the organism must be initially sensed and a signal transduced to the nucleus. Again, Fe will be sensed at both the local and systemic levels, but a master Fe sensor should link local response throughout the organism. A master Fe sensor must be able to bind Fe to sense cellular Fe stores and bind DNA to induce transcriptional changes. A well-described example of an Fe-sensing system is the yeast monothiol glutaredoxins (Gxs3/4) and Aft1/Aft2-sensing system [186,187,188,189]. When yeast is Fe sufficient, Gxs3/4 bind an Fe–S cluster, which allows the complex to bind the Fe transcription factors Aft1/Aft2 [190]. When Fe is limiting, the Fe–S cluster disassociates, thus freeing Aft1/Aft2 to induce transcriptional responses to Fe deficiency [191]. 

In eukaryotic photosynthetic organisms, a master Fe sensor has yet to be discovered, but there is evidence for Fe sensing components in Cyanobacteria. Fur, the Fe uptake transcription factor can both bind Fe and represses transcription (Figure 5a), for a review see [168]. Additionally, the Cyanobacterium *Synechocystis* sp. PCC 6803 Ferredoxin homologue, FdC2, contains an extended C terminus and truncated mutant versions of FdC2 inhibited the response to low Fe because the mutant could not upregulate the IsiA antenna that is critical for PSI protection during Fe deficiency responses (Figure 5a) [192]. FdC2 is predicted to bind an Fe–S cluster. The direct function of FdC2 in regulation of Fe deficiency is unclear but the Fe-binding ability along with changes in expression of the PSI protective protein, IsiA, makes it a strong candidate for Fe sensing. The FdC2 protein is conserved in green linages [192], suggesting that FdC2 may regulate Fe deficiency-dependent responses in other photosynthetic organisms.

In plants, potential candidate Fe sensors that are expressed at the root and shoot have been identified. The ubiquitin ligases, Brutus and Brutus-like (BTS/BTSL), that negatively regulate bHLH transcription factors in the Fe deficiency response, are also proposed to be local Fe sensors [184,185]. BTS and its rice orthologue (HRZ) both contain HHE domains that possibly bind Fe in vivo [183,193]. Further, BTS becomes destabilized when bound to Fe [183]. For BTS/BTSL a model is that during Fe replete conditions BTS and BTSL bind Fe and are destabilized. When Fe becomes limiting, BTS and BTSL cannot bind Fe, and mediate degradation of transcription factors necessary for upregulating the expression of proteins that mediate Fe deficiency responses [183]. Perhaps, the negative regulation by BTS/HRZ serves as a quick attenuation of the Fe deficiency response to protect against overaccumulation of Fe as the plant is attempting to increase Fe uptake. 

How might chloroplast Fe homeostasis be integrated into an Fe signaling mechanism? In the past, enzymes within the tetrapyrrole synthesis pathway have been suggested as viable candidates for chloroplast Fe sensing in plants because the tetrapyrrole pathway is known to have a component of retrograde signaling [194]. Several Genome Uncoupled (GUN) mutations are traced to proteins in the tetrapyrrole biosynthesis pathway [194]. GUN mutants are defective in communication between chloroplast and nucleus resulting in misexpression of nuclear encoded photosynthesis transcripts even when chloroplast development is inhibited [195]. As heme synthesis components and heme containing proteins seem to be less sensitive to Fe status compared to Fe–S-requiring proteins [90,126], it may be less likely that Fe deficiency induced retrograde signaling is initiated from heme biosynthesis. Because enzymes in the chlorophyll biosynthesis pathway are downregulated during acclimation to Fe limitation [124], it is possible that the chlorophyll branch of the tetrapyrrole pathway could be responsible for Fe status signaling. Mg-protoporphyrin IX, an intermediate in the chlorophyll biosynthesis pathway accumulates during Norflurazon (an inhibitor of carotenoid synthesis which is required to prevent bleaching of chlorophyll) treatment, and this uncouples plastid development from nuclear gene expression suggesting an interruption in retrograde signaling [196]. An accumulation of Mg-protoporphyrin IX during Fe deficiency resulting from downregulation of enzymes downstream of Mg-protoporphyrin IX production could provide a link between chlorophyll biosynthesis and Fe dependent retrograde signaling. Overall, the mechanism of retrograde signaling to induced Fe deficiency acclimation responses requires more attention [197].

The SUF Fe–S cluster assembly pathway may be a more viable candidate for Fe sensing. The SUF pathway has been linked with Fe homeostasis in other organisms, such as *E. coli* and the Cyanobacterium *Synechococcus* sp. PCC 7002, which specifically induce suf Fe–S assembly in response to oxidative stress and Fe deficiency [47,148]. Further, the Cyanobacterial SufA and IscA are proposed to regulate Fe–S production in response to Fe limitation. It is proposed that IscA and SufA have regulatory roles in Fe–S cluster production as knockout mutants of iscA and sufA had increased expression of the *suf* operon in Fe limiting conditions [148]. In addition, the Cyanobacterium *Synechocystis* sp. PCC 6803 suf scaffold was repressed under Fe replete conditions by the Fe–S-binding transcriptional repressor, SufR [198,199], suggesting that regulation of suf Fe–S cluster assembly to avoid excess Fe–S clusters is important. In higher plants, the SUF system is also sensitive to low Fe, specifically in the downregulation of SUFB. While SUFB has been proposed as possible Fe sensor in Arabidopsis [200], its direct role in regulation and Fe binding has not been studied. 

New molecular details underlying systemic Fe-signaling mechanisms have been recently uncovered in plants. It has long been demonstrated through grafting and split root experiments that a Fe deficiency signal is initiated from the shoots to upregulate root Fe uptake [201,202]. Recently, phloem Fe homeostasis has been linked to upregulation of Fe uptake. Oligopeptide Transporter 3 (OPT3) is predicted to load Fe into the shoot phloem from the xylem during Fe deficiency (Figure 3c) [203]. A knockout mutant of OPT3 displayed low phloem Fe and a constitutive upregulation of Fe uptake. Alternatively, knockout mutants of two Yellow Stripe Like proteins, Yellow Stripe Like 1 and 3 (YSL1/YSL3), could not induce expression of root Fe uptake systems during Fe limitation [204]. Phloem signaling peptides, Ironman/Fe Uptake Inducing Peptide (IMA/FEP) are highly expressed early in the Fe deficiency response, and over expression of IMA3/FEP1 resulted in increased expression of bHLH039 and upregulation of root Fe uptake [205,206]. Interestingly, IMA/FEP peptides contain a highly conserved aspartate domain that may bind Fe. 

## 6. Regulating Acclimation to Excess Fe 

To avoid Fe excess, plants tightly regulate Fe uptake at the root, and employ mechanisms of sequestration within the cell [18]. This regulation of Fe uptake along with the poor bioavailability of Fe in soil [24], makes superoptimal availability of Fe a less likely occurrence in nature compared to Fe deficiency. However, in some cases, such as anoxic soil conditions, Fe may be more available to plants resulting in increased uptake and Fe toxicity. Here we briefly discuss regulation of responses to excess Fe with a focus on ferritin expression. For a more comprehensive review of this topic, see [116]. In Fe excess conditions, transcription of FER1 increases, but so does mRNA turnover, perhaps as a mechanism to quickly modulate the amount of functional FER1 in the chloroplast so that enough Fe is available for metabolism [207]. ILR3 represses FER transcription during Fe deficiency, but this repression is alleviated during Fe excess conditions, thus allowing for FER accumulation [178]. FER regulation may also be important in switching responses between Fe deficiency and resupply. Upon Fe resupply, a burst of Nitric Oxide (NO) is necessary for the FER1 promoter to become de-repressed [116].

## 7. Conclusions 

As the chloroplast is a major sink for Fe in a plant, it is vital to understand how the chloroplast uses Fe and how it responds to deficiency to maintain photosynthesis. Insights from Fe metabolism of other photosynthetic organisms may translate to the study of chloroplast Fe deficiency regulation. Photosynthetic organisms share the Fe deficiency responses of decreased photosynthetic output and Suf Fe–S cluster assembly. Our understanding of regulation of the chloroplast acclimation to Fe deficiency is limited. There is no enrichment of bHLH cis elements in the promoters of the early-responding Fd2 and SUFB transcripts in Arabidopsis [126]. ERF transcription factors may be important for regulation of chloroplast Fe deficiency responses possibly through the PAP/SAL1 retrograde signaling pathway [167]. To clearly determine possible transcription factors that may regulate chloroplast acclimation to low Fe, requires an analysis of all the promoters of transcripts that are regulated, very early after Fe limitation is induced. Most likely, the acclimation of the chloroplast to Fe deficiency will be regulated by multiple transcriptional, post-transcriptional, and post-translational mechanisms, as observed in Cyanobacteria. 

Fe–S clusters are required for many photosynthetic electron transport proteins. It can be envisioned that the decrease in Fe–S assimilation in low Fe conditions due to the downregulation of SUFB may produce a signal to the nucleus to further downregulate transcripts encoding photosynthetic Fe–S proteins, such as Fd2 and Rieske protein in Arabidopsis. Moreover, it is possible that an imbalance in the photosynthetic electron transport machinery activates operational signals to the nucleus [197] in order to adjust expression of photosynthetic Fe proteins. However, upon Fe deficiency induction in Arabidopsis, transcripts encoding photosynthetic proteins are downregulated much later when compared to the key Fe–S cluster assembly factor SUFB [126] which argues against a direct link between Fe–S assembly and regulation of photosynthetic gene expression.

Research in plant Fe deficiency over the past decades has determined how Fe proteins respond to Fe deficiency in the chloroplast and has identified key regulatory factors in root Fe uptake and Fe sequestration. Now, questions that remain are centered around how the leaf Fe deficiency response is regulated and how the plant coordinates its leaf and root Fe deficiency responses. By understanding the regulatory pathways that produce the Fe economy response, eventually we can work backwards to determine the chloroplast Fe sensor, and perhaps the master Fe sensor, that is initiating a signal to alter expression of photosynthetic genes. Could chloroplast responses like downregulation of SUFB and Fd2 be regulated by the same families of transcription factors as the roots? Clearly, phloem-mediated Fe signaling is required to coordinate shoot Fe needs with root uptake. How the leaf cell communicates Fe needs that lead to upregulation of phloem Fe signaling, and whether or not chloroplast Fe status is integrated into this communication, are exciting topics for further study.

## Figures and Tables

**Table 1:** *Arabidopsis thaliana* chloroplast Fe homeostasis genes and their *Chlamydomonas reinhardtii* and *Synechocystis* PCC 6803 (Cyanobacterial) orthologues. Arabidopsis protein name and ID are listed for plastid Fe homeostasis proteins discussed in this review. Studies that identified Chlamydomonas and Cyanobacterial orthologues for these proteins are referenced next to the gene name. In cases where an orthologue has not been previously identified in the literature, the Arabidopsis protein sequence was used as a query sequence in NCBI BLAST. Orthologues were determined if the Chlamydomonas and Cyanobacterial sequences had at least 20% sequence similarity for at least 50% of the query sequence. When possible, Cyanobacterial gene names are listed for *Synechocystis* PCC 6803. Otherwise, the NCBI gene ID is listed.

Function	*Arabidopsis* Gene Name	*A. thaliana*	*C. reinhardtii*	*Synechocyst* Is PCC 6803
Transport	FRO7	AT5G49740	Cre04.g227400.t1.2	
	PIC1	AT2G15290	Cre10.g454734.t2.1	sll1656 [103]
	ATP-Binding Cassette I11 (ABCI11)/NAP14	AT5G14100	PNW71978/Cre16.g687550.t1.1 [107]	slr0354 [107]
	ABCI10	AT4G33460	XP_001703542/Cre03.g164150.t1.1 [107]	sll1623 [107]
	ABCI12	AT3G21580	PNW75614/Cre12.g533950.t1.1 [107]	slr1978 [107]
	Multiple Antibiotic Resistance 1 (MAR1)	AT5G26820	Cre03.g175200.t1.2	lap75
	Yellow Stripe Like 4/6 (YSL4/6)	(4) AT5G41000, (6) AT3G27020		
ROS homeostasis	Fe SuperOxide Dismutase (FSD)	AT4G25100 AT5G51100 AT5G23310	Cre10.g436050.t1.2	WP_010872652
	Stromal Ascorbate Peroxidase (sAPX)	AT4G08390	Cre02.g087700.t1.2	
	Conserved in Green Linage and Diatoms 27 (CGLD27)	AT5G67370	Cre05.g237050.t1.1	WP_010873853
Suf Fe–S assembly	SUFB	AT4G04770	Cre15.g643600.t1.2 [128]	slr0074 [128]
	SUFC	AT3G10670	Cre07.g339700.t1.2 [128]	slr0075 [128]
	SUFD	AT5G44316	Cre12.g513950.t1.2 * [128]	slr0076 [128]
	SUFS	AT1G08490	Cre12.g525650.t1.2 [128]	slr0077 [128]
	SUFE	AT4G26500	Cre06.g309717.t1.1 [128]	slr1419 [128]
	SUFA	AT1G10500	Cre07.g349600.t1.2 [128]	slr1417 [128]
	Nitrogen Fixation U-Like (NFU)	(1) AT4G01940 (2) AT5G49940 (3) AT4G25910	(1) Cre18.g748447.t1.1 (2) Cre12.g504150.t2.1 (3) Cre17.g710800.t2.1 [128]	ssl2667 [128]
	High Chlorophyll Fluorescence 101 (HCF101)	At3g24430	Cre01.g045902.t1.1 [128]	slr0067 [128]
	Monothiol Glutaredoxins (GRXS)	(14) AT3G54900 (16) AT2G38270	(14) Cre07.g325743.t1.1 (16) Cre01.g047800.t1.1 [128]	WP_010871706
	NEET	AT5G51720	Cre01.g050550.t1.2	
Heme biosynthesis	Ferrochelatase (FC)	AT5G26030 AT2G30390	Cre07.g339750.t2.1	WP_010873751
	Sirohydrochlorin Ferrochelatase B (SirB)		Cre04.g214100.t1.2	WP_010874018
Sequestration	Ferritin (FER)	(1) AT5G01600 (3) AT3G56090 (4) AT2G40300	Cre09.g387800.t1.2, Cre13.g574500.t1.1	sll1341 ** [129] slr1890 ** slr1894 ** [130]

* *C. reinhardtii* SUFD was more similar to *A. thaliana* SufB. The *C. reinhardtii* gene ID listed is for the annotated SUFD gene; ** No sequence similarity was found for *A. thaliana* ferritin in *Synechocystis* PCC 6803. Genes listed are for Bacterioferritin A and B, and MrgA.
